# Changes in nitrogen and phosphorus availability driven by secondary succession in temperate forests shape soil fungal communities and function

**DOI:** 10.1002/ece3.10593

**Published:** 2023-10-09

**Authors:** Xinze Geng, Jincheng Zuo, Yunhao Meng, Yanhui Zhuge, Ping Zhu, Nan Wu, Xinfu Bai, Guangyan Ni, Yuping Hou

**Affiliations:** ^1^ College of Life Sciences Ludong University Yantai China; ^2^ School of Resources and Environmental Engineering Ludong University Yantai China; ^3^ Key Laboratory of Vegetation Restoration and Management of Degraded Ecosystems and Guangdong Provincial Key Laboratory of Applied Botany, South China Botanical Garden Chinese Academy of Sciences Guangzhou China

**Keywords:** community potential function, ectomycorrhizal, fungal community structure, fungal pathogen, secondary succession, temperate forest

## Abstract

The soil fungal community plays an important role in forest ecosystems and is crucially influenced by forest secondary succession. However, the driving factors of fungal community and function during temperate forest succession and their potential impact on succession processes remain poorly understood. In this study, we investigated the dynamics of the soil fungal community in three temperate forest secondary successional stages (shrublands, coniferous forests, and deciduous broad‐leaved forests) using high‐throughput DNA sequencing coupled with functional prediction via the FUNGuild database. We found that fungal community richness, α‐diversity, and evenness decreased significantly during the succession process. Soil available phosphorus and nitrate nitrogen decreased significantly after initial succession occurred, and redundancy analysis showed that both were significant predictors of soil fungal community structure. Among functional groups, fungal saprotrophs and pathotrophs represented by plant pathogens were significantly enriched in the early‐successional stage, while fungal symbiotrophs represented by ectomycorrhiza were significantly increased in the late‐successional stage. The abundance of both saprotroph and pathotroph fungal guilds was positively correlated with soil nitrate nitrogen and available phosphorus content. Ectomycorrhizal fungi were negatively correlated with nitrate nitrogen and available phosphorus content and positively correlated with ammonium nitrogen content. These results indicate that the dynamics of fungal community and function reflected the changes in nitrogen and phosphorus availability caused by the secondary succession in temperate forests. The fungal plant pathogen accumulated in the early‐successional stage and ectomycorrhizal fungi accumulated in the late‐successional stage may have a potential role in promoting forest succession. These findings contribute to a better understanding of the response of soil fungal communities to secondary forest succession and highlight the importance of fungal communities during the successional process.

## Graphical abstract

The driving factors of fungal community and function during temperate forest succession and their potential impact on succession processes are poorly understood. In this study, we investigated the dynamics of the soil fungal community in three temperate forest secondary successional stages. Our results indicated that the dynamics of fungal community and function reflected the changes in soil nitrate nitrogen and available phosphorus content caused by the secondary succession of temperate forests.

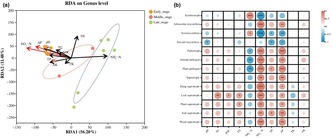

## 1 INTRODUCTION

Forest ecosystems play an important role in global biochemical cycles, but natural disturbances and human activities have resulted in the expansion of secondary forest succession, which has become more common on a global scale (Chazdon et al., 2016; Millar & Stephenson, 2015; Pugh et al., 2019). It is therefore becoming increasingly important to investigate secondary forest succession in temperate forest ecosystems. Forest succession can significantly affect the composition and function of soil fungal communities (Cline & Zak, 2015; Zhou et al., 2017). Since they constitute important participants in ecosystem soil processes, changes in the function of fungi may exert feedback on aboveground vegetation and thereby affect succession processes (Geisen et al., 2022; Knoblochová et al., 2017; Liao et al., 2018). However, much remains unknown about the changing patterns of fungal communities during temperate forest succession and their potential impacts.

Forest succession can indirectly affect soil properties by changing the input of plant litter and root exudates, and by significantly affecting soil microbial community structure and function. This effect has been widely demonstrated in studies on bacterial communities (Dai et al., 2021; Zhang, Wang, et al., 2022). Soil pH, soil organic carbon (SOC), soil total nitrogen (TN), soil available nitrogen (AN), and soil available phosphorus (AP) exert significant influences on bacterial communities during secondary forest succession (Dai et al., 2021; Qu et al., 2020; Zhang et al., 2021). Qu et al. (2020) found that the increase in soil pH during forest secondary succession changed the structure of soil bacterial community and caused the dominant functional groups involved in the carbon cycle to be replaced by the groups involved in the nitrogen and sulfur cycles. The increase in soil carbon and nitrogen contents during secondary succession resulted in a significant decrease in the abundance of nitrification, and aerobic ammonia oxidation bacteria adapted to the oligotrophic environment had a considerable impact on nutrient cycling in forest soil (Zhang et al., 2023). Compared with bacteria, soil fungi are more sensitive to forest secondary succession, which is often accompanied by changes in the soil microbial community from bacteria‐ to fungi‐dominated (He et al., 2022; Li et al., 2022; Susyan et al., 2011). In forest ecosystems, the effect of the soil fungal community on ecosystem function is stronger than that of bacteria (Jiang et al., 2021). However, knowledge of the effects of soil properties on the structure and function of soil fungal communities’ change during the succession of secondary forests is limited. Therefore, further research is needed to deepen our understanding of the driving factors of the soil microbial community in secondary succession of temperate forests.

Soil fungal pathogens and mutualists are key driving factors of plant community succession (Nara & Hogetsu, 2004; Richard et al., 2009; Van Der Putten & Peters, 1997). The accumulation of soil fungal pathogens during the early succession of dune vegetation significantly inhibited the growth of conspecific seedlings, promoting the replacement of early‐ by late‐successional species (Van Der Putten et al., 1993). Soil fungal pathogens can significantly increase seedling mortality near their parent trees and could be an important contributor to tropical forest population regulation (Bagchi et al., 2010; Liang et al., 2016). Symbiotic fungi can also influence the succession process through positive feedback with plants (Nara, 2006; Roy‐Bolduc et al., 2016). The accumulation of ectomycorrhizal and arbuscular mycorrhizal fungi is an important mechanism for host species to establish monodominant communities (Bennett et al., 2017; Laliberté et al., 2015; Liu et al., 2021). Although soil fungi thus play an important role in plant community dynamics, much of the potential impact of functional changes of fungal communities on temperate forest succession remains yet to be elucidated.

This study was conducted on Kunyu Mountain, a temperate forest nature reserve located in northern China. We selected three typical forest types representing the succession process, namely shrublands, coniferous forests, and deciduous broad‐leaved forests. We used high‐throughput sequencing techniques combined with fungal functional taxon prediction (FUNGuild version 1.0) for the analysis. We aimed to answer the following questions: (1) How do the soil fungal communities and function structure respond to temperate forest succession? (2) What are the potential impacts of changes in soil fungal community function on forest succession processes?

## 2 MATERIALS AND METHODS

### 2.1 Study area

The study sites were located in Kunyu Mountain Nature Reserve, Shandong, China (121°3′70″‐121°51′0″ E, 37°12′20″–37°18′50″ E) in a warm temperate continental monsoon climate, with an annual average temperature of 11.9°C, and annual rainfall of 650–900 mm (Wang et al., 2009). The soil in the study area is classified as Eutric Cambisols according to the scheme of the Food and Agriculture Organization (FAO).

The main plant communities in the study area were natural *Pinus densiflora* forests, scrub and meadows, and deciduous broad‐leaved forests dominated by oak species. In the 21st century, the native *Pinus densiflora* forests have been destroyed by insect pests. The selected area was closed for an extended period as a former red pine logging site, which led to randomly distributed forest patches recovering from the soil seed bank in some unmanaged sites (Sun et al., 2011). This process forms a typical natural forest community which can represent the early‐, middle‐, and late‐successional stages of secondary succession. We selected the most representative plant communities in the three secondary successional stages of the forest on Kunyu Mountain based on the descriptions in *Vegetation of Shandong* (Wang & Zhou, 2000). These were shrubs (*Grewia biloba* G. Don, *Rhus chinensis* Mill.) in the early‐successional stage, coniferous forest (*Pinus densiflora* Sieb. et Zucc.) in the middle‐successional stage, and oak deciduous broad‐leaved forest (*Quercus acutissima* Carr., *Quercus variabilis* Blume) in the late‐successional stage.

### 2.2 Sample collection

The soil samples were collected on April 28, 2021. Based on similar conditions in elevation, topography, and understory vegetation, we selected three monodominant forest stands for each forest type, each with a size of 100 × 100 m. Five individuals with similar diameter at breast height were chosen from each stand. Soil samples were collected using sterilized shovels and resterilized after sampling a forest stand (soaked in 95% ethanol for 30 s). Three soil subsamples were collected at a depth of 0–10 cm after litter removal, observing 1 m distance from the central tree in three directions at 120° angles. After the visible stones and roots were removed, all five individual subsamples were homogenized to a single sample (Qu et al., 2022). Care was taken during tree selection to maintain at least a 10 m distance from nontarget tree species, and each targeted tree was located farther than 20 m from the forest edge. A distance of at least 10 m was maintained between sampled trees to ensure spatial independence.

All samples were put on ice and transported back to the laboratory. After removing impurities (stones and roots), each sample was divided into three subsamples. (1) One subsample was immediately air‐dried for determining soil pH, TN, total carbon (TC), total phosphorus (TP), total potassium (TK), soil organic carbon (SOC), available phosphorus (AP), and soil available potassium (AK). (2) A −20°C storage subsample was used to analyze nitrate nitrogen (NO_3_^−^‐N) and ammonium nitrogen (NH_4_^+^‐N) within 2 weeks. (3) A −80°C storage subsample was used to extract DNA.

### 2.3 Soil chemical analysis

Soil chemical analysis was performed following previously described methods (Bao, 2000). Soil pH was measured using an electrode pH meter in 1:2.5 (w/v) soil water suspensions (Carter & Gregorich, 2007). Soil total carbon and nitrogen were determined using dry combustion with an elemental analyzer (Vario EL; Elementar Analysensysteme). The SOC concentration was measured using potassium dichromate dilution calorimetry (Walkley, 1947). The NO_3_^−^‐N and NH_4_^+^‐N contents were determined after extraction with KCl solution using a flow analyzer (A16786; Alliance Instruments). To measure TP and TK, the samples were first digested with sodium hydroxide in a muffle furnace (450°C). TP and TK were then determined using a spectrophotometer and a flame photometry detector, respectively (Tedersoo et al., 2015; Wu et al., 2019). AP content was determined using the sodium bicarbonate extraction–molybdenum‐antimony colorimetric method (Wei et al., 2020). AK content was determined using the ammonium acetate extraction–flame photometry method (Meng et al., 2022).

### 2.4 DNA extraction and high‐throughput DNA sequencing

We isolated the total genomic DNA from 250 mg of each homogenized soil sample using an EZNA® Soil DNA Kit (Omega Bio‐tek) and quantified concentrations in a NanoDrop2000 (Thermo Fisher Scientific). The primers ITS1/ITS4 were used to amplify the ITS1 region (Lekberg et al., 2021; White et al., 1990). The purified amplicons were pooled in equimolar ratios, and paired‐end sequencing was performed using the Illumina MiSeq PE300/NovaSeq PE250 platform (Illumina) because Illumina MiSeq has a higher throughput and lower error rate than other high‐throughput sequencing instruments (Frey et al., 2014; Loman et al., 2012). The sequences were processed using Mothur software (version 1.44.3) following the standard operating procedure (SOP) with some modifications (Qu et al., 2020; Schloss et al., 2009). Putative chimeras (using Mothur's UCHIME) were screened out if these sequences contained ambiguous (N) bases, homopolymers longer than 8 BP, mean mass score less than 25, and less than 250 BP. The high‐quality and unique sequences were classified against the UNITE database (version 8) with a bootstrap cutoff of 80 (Abarenkov et al., 2010). The “remove lineage” command removed nonfungal sequences (Tedersoo et al., 2010). We used the FUNGuild database to assign ecological guilds to OTUs and only retained confidence rankings of “probable” or “highly probable” guilds (Nguyen et al., 2016). This database offers a consistent and straightforward method for classifying large sequence libraries into ecologically relevant classes and has been widely used in soil ecology research (Liang et al., 2016; Ramirez et al., 2019; Zhang et al., 2020).

### 2.5 Statistical analysis

The Chao1 index, Shannon evenness index, and Shannon–Wiener index were used as diversity estimates. One‐way analysis of variance (ANOVA) and false discovery rate correction were used to evaluate the differences in the soil nutrients, α‐diversity, fungal community function, and the relative abundance of the dominant phyla and genera among different forest types in the succession stage. Nonmetric multidimensional scaling (NMDS) plots were used to represent the relationships between fungal community compositions in different forest types. Their successional stages were prepared using Bray–Curtis distances obtained with the “vegan” package in R. Subsequently, Adonis (999 permutations) was performed in R to evaluate the significant differences in soil fungal community composition among successional stages. Redundancy analysis (RDA) was used to evaluate the relationships between the soil fungal community and soil nutrients. Spearman analysis was used to analyze the correlation between the sequence abundance of fungal functional groups and soil nutrients. All statistical analyses were performed with R program version 4.2.1 (R Core Team, 2022). Differences with *p* < .05 were regarded as statistically significant.

## 3 RESULTS

### 3.1 Soil properties and fungal α‐diversity during forest succession

**FIGURE 1:**
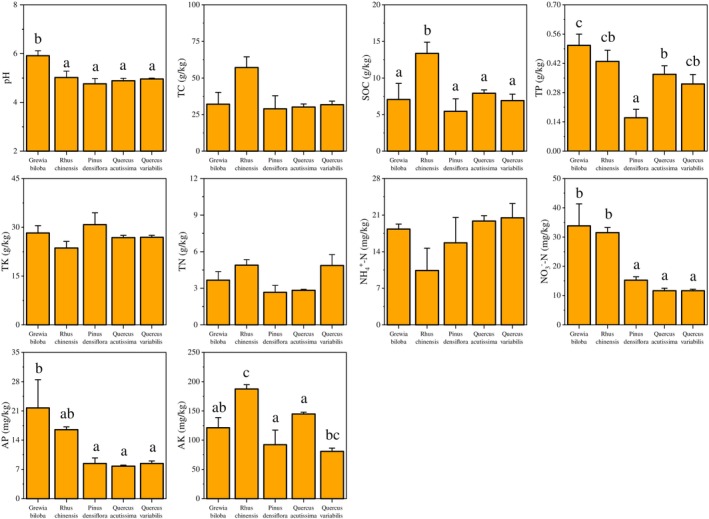
Soil properties during the forest succession process. Different letters indicate significant differences (*p* < .05), no letters indicate no significant differences, and the error bars represent the standard error. AK, available potassium; AP, available phosphorus; NH_4_^+^, ammonium nitrogen; NO_3_^−^, nitrate nitrogen; SOC, soil organic carbon; TC, total carbon; TK, total potassium; TN, total nitrogen; TP, total phosphorus.

There were differences in soil pH, SOC, TP, TN, AP, and AK contents among the forest sites representing different successional stages (Figure 1). Soil NO_3_^−^‐N was significantly higher in the forest type of early‐successional stage than in other stages of succession (*p* < .01). Soil pH and AP were highest in *G. biloba* forest at the early‐successional stage and significantly higher than those in middle‐ and late‐stage forest types (*p* < .05, Figure 1; Table S1). TP in the middle‐stage forest was significantly lower than at other stages of succession (*p* < .01). SOC and AK content was significantly enriched in the soil of *R. chinensis* forest in early succession (*p* < .05, Figure 1).

**FIGURE 2:**
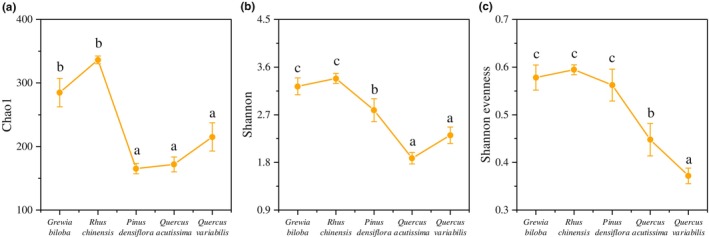
Fungal community richness (a), α‐diversity (b), and Shannon evenness (c) during the forest succession process. Different letters indicate significant differences (*p* < .05), no letters indicate no significant differences, and the error bars represent the standard error.

The secondary successional stage significantly altered the fungal community richness (Chao1), diversity (Shannon), and evenness (Shannon evenness). All three indicators showed a trend of decrease with succession (Figure 2). The richness index was highest in the forest types in the early‐successional stage and significantly higher than in the middle‐ and late‐successional stages (*p* < .01, Figure 2a; Table S2). The Shannon index decreased significantly during forest succession (*p* < .01, Figure 2b). The evenness index decreased in the late‐successional stage, when it was significantly lower than in the early‐ and middle‐successional stages (*p* < .01, Figure 2c).

### 3.2 Fungal community composition during forest succession

**FIGURE 3:**
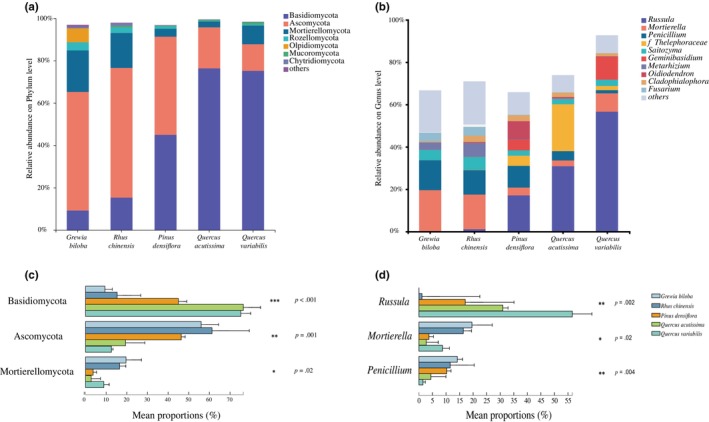
The composition and differences of fungal communities at the phylum (a,c) and genus levels (b,d) during forest succession.

All sequences (1,194,454) were classified according to fungal phylum and were assigned to 2994 OTUs across all samples. These OTUs belonged to 15 phyla, including 63 classes, 151 orders, and 667 genera. The most abundant phyla across all samples were Basidiomycota (44.27% of sequences), followed by Ascomycota (39.11%), Mortierellomycota (10.28%), Rozellomycota (1.63%), and Olpidiomycota (1.34%; Figure 3a). The abundance of some phyla differed during the succession process, but differences were not significant between forest types at the same successional stage. The abundance of Basidiomycota increased significantly (*p* < .001, Table S3) during secondary succession, whereas Ascomycota abundance was significantly lower (*p* < .001) in the late‐successional stages. Mortierellomycota abundance was the highest in the early‐successional stage and decreased significantly after succession occurred (Figure 3c).

**FIGURE 4:**
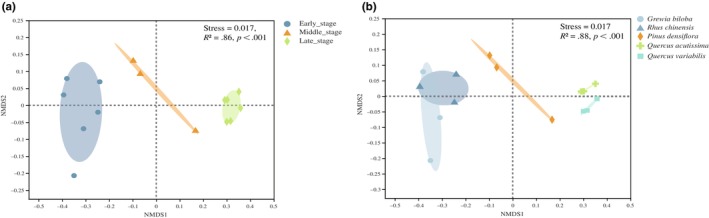
Nonmetric multidimensional scaling (NMDS) of fungal community composition between different successional stages (a) and different forest types (b).

The most abundant genus was *Russula* (Basidiomycota, 21.15% of sequences) followed by *Mortierella* (Mortierellomycota, 10.2%), *Penicillium* (Ascomycota, 8.37%), and *Saitozyma* (Basidiomycota, 3.79%). A detailed genus list is shown in Figure 3b. *Russula* abundance increased with succession and was significantly higher in the late‐successional *Q. acutissima* forest than in other forest types except the *Q. variabilis* forest (*p* < .01). The abundance of *Mortierella* was highest in the early‐successional shrublands and significantly higher than in *P. densiflora* and *Q. acutissima* forest (*p* < .05). The abundance of *Penicillium* was significantly lower in the late‐successional stage (*p* < .01, Figure 3d; Table S3). NMDS based on Bray–Curtis distances shows that soil fungal communities were significantly separated between different forest types and different successional stages. Subsequent Adonis analysis confirmed the differences among the communities (*p* < .05 for each pair, Figure 4).

### 3.3 Fungal functional groups during forest succession

Based on the annotation of fungal ITS sequences in the FUNGuild database, the fungal communities were classified into 17 guilds, which belonged to three trophic modes: symbiotroph, pathotroph, and saprotroph. The relative abundance of trophic modes differed significantly among forest types representing different successional stages. Fungal symbiotrophs were significantly enriched in the late‐successional stage (*p* < .01), while fungal pathotrophs were significantly enriched in the early stage (*p* < .01). Fungal saprotroph abundance was significantly lower in the *Q. variabilis* forest at the late‐successional stage than at the early‐successional stage (*p* < .05, Figure S1, Table S4).

**FIGURE 5:**
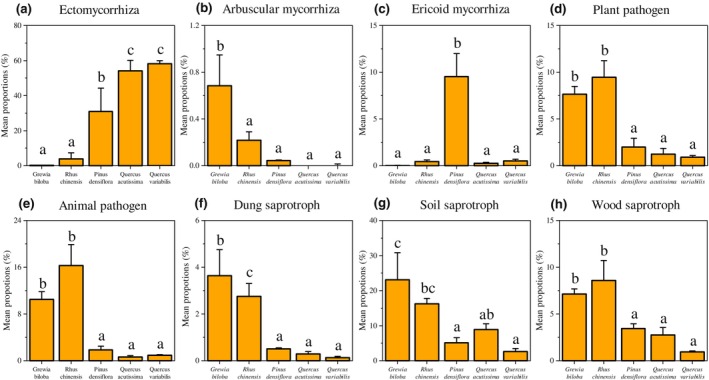
Bar chart showing the significantly different functional guilds during the forest succession. Different letters indicate significant differences (*p* < .05), no letters indicate no significant differences, and the error bars represent the standard error.

Among the symbiotrophic guilds, ectomycorrhizal fungi had the highest relative abundance in the later stages (*p* < .01) and showed an increasing trend during forest succession (Figure 5). In contrast, the highest relative abundance of arbuscular mycorrhizal fungi in the early‐successional stage was found in *G. biloba* forest (*p* < .01). The relative abundance of ericoid mycorrhiza increased significantly in the middle‐successional stage (*p* < .01). Within the pathotrophic guilds, animal and plant pathogens were significantly more abundant in the early‐successional stage than in the middle‐ and late‐successional stage (*p* < .01); however, there was no significant difference between the middle and late stages (Figure 5). The distribution of soil, leaf, and wood saprotrophs was significantly different among fungal saprotrophs. All three groups were significantly enriched in the early‐successional stage (*p* < .01, Figure 5; Table S4).

### 3.4 Relationships between soil chemical properties, fungal community composition, and functional groups

**FIGURE 6:**
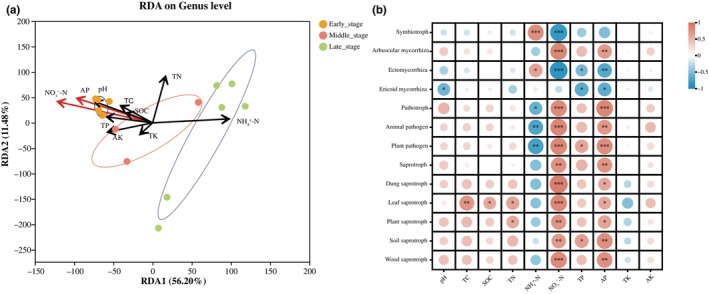
Redundancy analysis (RDA) biplot integrating soil nutrients under different successional stages. (a) Parameters represented by longer arrows made a stronger contribution to group separation, red arrows represent a significant effect, and black arrows represent no significant effect. (b) Double matrix correlation heatmap of differential fungal functional guilds and environmental factors with a significant difference. The color key represents the range of different *R* values. Red represents positive correlation, and blue represents negative correlation. *, ** and ***Indicate significant differences at .01 < *p* < .05, .001 < *p* < .01, and *p* < .00. AK, available potassium; AP, available phosphorus; NH_4_^+^‐N, ammonium nitrogen; NO_3_^−^‐N, nitrate nitrogen; SOC, soil organic carbon; TC, total carbon; TK, total potassium; TN, total nitrogen; TP, total phosphorus.

The first and second axes of the RDA analysis explained 55.74% and 10.88% of the variation in fungal genus‐level communities, respectively (66.62% combined; Figure 6a). The results indicate that soil NO_3_^−^‐N (*r* ^2^ = .66, *p* < .01) and AP (*r* ^2^ = .46, *p* < .05) were the most significant soil nutrient factors affecting fungal community composition. Both were positively correlated with the fungal community composition in the early‐successional stage and negatively correlated with the fungal community in the middle‐ and late‐successional stages. This effectively explained the separation of fungal communities in the three successional stages (Figure 6a).

Correlation analysis highlighted different relationships between fungal functional guilds and soil nutrients (Figure 6b). All saprotrophic guilds were significantly positively correlated with NO_3_^−^‐N and AP. Leaf saprotrophs were significantly and positively correlated with TC, SOC, and TN; plant saprotrophs with TN; and soil saprotrophs with TP (Figure 6b). All pathotrophic guilds were significantly positively correlated with NO_3_^−^‐N and AP and were significantly negatively correlated with NH_4_^+^‐N. Plant pathogens were significantly positively correlated with TP (Figure 6b). Symbiotrophs were significantly positively correlated with NH_4_^+^‐N and negatively correlated with NO_3_^−^‐N. Arbuscular mycorrhizal fungi were significantly positively correlated with NO_3_^−^‐N and AP. Meanwhile, ectomycorrhizal fungi were significantly positively correlated with NH_4_^+^‐N and negatively correlated with NO_3_^−^‐N, TP, and AP. Ericoid mycorrhiza was significantly negatively correlated with pH, TP, and AP (Figure 6b).

## 4 DISCUSSION

This study illustrates the response pattern of fungal communities during the secondary succession of temperate forests, from shrublands and coniferous forests to deciduous broad‐leaved forests. The change in soil properties caused by succession was likely the driver behind the changes in the fungal community. Soil AP and NO_3_^−^‐N were strongly associated with fungal community structure and function. In the early stage of succession, the shrub biomes significantly accumulated fungal pathogens, while in the late stage, the deciduous broad‐leaved forest significantly accumulated ectomycorrhizal fungi. The changes in fungal community function may have a potential influence on the succession process of temperate forests.

We found that secondary succession in temperate forests had significant effects on soil nutrients, with AP and NO_3_^−^‐N being significantly higher in early‐ than in later‐successional stages. Nitrogen and phosphorus availability showed a decreasing trend during forest succession. In previous studies on subtropical and tropical forest ecosystems, succession was often accompanied by an increase in soil nutrient availability (Bauters et al., 2022; Sullivan et al., 2019; Zheng et al., 2020). In contrast, in temperate forest ecosystems, plants in the middle‐ and late‐successional stages were predominantly ectomycorrhizal trees from the Pinaceae and Fagaceae families. These are characterized by slowly decomposing litter and lower nutrient cycling rates, which may explain the reduced availability of nitrogen and phosphorus during temperate forest succession (Lin et al., 2017; Phillips et al., 2013; Schilling et al., 2016). The tendency for fungal community diversity to decrease across successional stages may be explained by changes in soil nutrients (Dini‐Andreote et al., 2015; Zheng & Song, 2022). In the early stages of secondary succession, disturbances often result in the liberation of nutrients. This can facilitate the rapid colonization of soils by a diverse microbial population (Zhang et al., 2016). Plants appearing during the secondary stage of succession have been shown to grow faster and consume more nutrients (Lajtha, 2019). As succession proceeds, the use of nutrients by plants can therefore lead to a decrease in soil nutrient availability (Huang et al., 2012), thereby reducing the diversity of the soil fungal community.

NO_3_^−^‐N and AP were the main predictors of soil fungal community structure during this succession process and were significantly correlated with fungal functional groups. As important decomposers of soil organic matter, saprophytic fungi are sensitive to changes in soil properties. The abundance of saprotrophic guilds was positively correlated with NO_3_^−^‐N and AP, and the high abundance of saprotrophs led to a faster turnover of soil nutrients and facilitated the maintenance of high nutrient availability (Liu et al., 2022; Wu et al., 2019). High nitrogen and phosphorus availability also promoted the colonization of fungal saprotrophs in the soil (Zhang, Dong, et al., 2022). Different guilds of saprotrophs showed different responses to total nutrients. This may reflect variation in the preference of different saprotroph guilds in temperate forest soils for total nutrients in the substrate (Cao et al., 2022; Cline et al., 2018; Guo et al., 2022).

Furthermore, NO_3_^−^‐N and AP contents during the succession process were not only negatively correlated with the abundance of ectomycorrhizal fungi but also positively correlated with those of arbuscular mycorrhizal fungi. This can be explained by the difference in plant nutrient acquisition between these two groups (Cheeke et al., 2016; Genre et al., 2020; Tedersoo et al., 2012). Ectomycorrhizal fungi have the ability to access nitrogen and phosphorus from organic material and transfer them to the host plant (Smith & Read, 2008; Tedersoo & Bahram, 2019), whereas arbuscular mycorrhizal fungi have relatively limited capacity for enzymatic degradation and mainly take up nutrients in mineral form (Chen et al., 2018; Tisserant et al., 2013). Therefore, soils with high nitrogen and phosphorus availability are more conducive to colonization by arbuscular mycorrhizal fungi. Given that ectomycorrhizal fungi incur a considerable carbon cost from cooperation, host plants in high nutrient availability environments often reduce their symbiosis with these fungi (Guo et al., 2021; Nilsson et al., 2005; Peng et al., 2022). Ectomycorrhizal fungi are thus more abundant in nutrient‐deficient than in nutrient‐rich environments (Bai et al., 2019). This phenomenon is consistent with the findings of our study.

One emerging pattern in natural systems analysis is that pathogens often thrive in resource‐rich environments (Revillini et al., 2016; Reynolds et al., 2003). A series of studies in grassland ecosystems and agroecosystems have also shown that high nitrogen and phosphorus availability often leads to an increase in fungal pathogens (Ebeling et al., 2021; Lekberg et al., 2021). We observed that the abundance of pathotrophic guilds was positively correlated with NO_3_^−^‐N and AP. This suggests that higher soil nutrient availability may be the reason for the accumulation of fungal pathogens in the early stage of succession. However, the correlation between plant pathogen fungi and soil nutrients is the opposite of that observed in ectomycorrhizal fungi, as the association of plant roots with ectomycorrhizal fungi also protects the host from pathogens (Bennett et al., 2017; Liang et al., 2020; Tedersoo et al., 2020). The correlation between plant pathogens and soil nutrients may thus be indirectly driven by ectomycorrhizal fungi.

We found that during secondary succession in temperate forests, early‐successional forest types accumulated fungal plant pathogens, while late‐successional forest types accumulated ectomycorrhizal fungi. The positive driving role of microbial pathogens in community succession and the accumulation of pathogenic fungi in the soil can cause reduced seedling recruitment and survival around a conspecific adult. This in turn may facilitate forest succession (Domínguez‐Begines et al., 2020; Van Der Putten et al., 1993). The accumulation of ectomycorrhizal fungi is an important mechanism for maintaining dominant communities of host trees in temperate forests (Chen et al., 2019; Liang et al., 2020). As an important plant symbiont, ectomycorrhizal fungi can expand the absorption range of the root system and transport nutrients from organic matter to the host (Genre et al., 2020; Tedersoo et al., 2020). Given that we observed that nutrient availability decreased with succession, ectomycorrhizal fungi may play a more important role in nutrient acquisition in late‐successional forest types. We also observed that the relative abundance of ectomycorrhizal fungi increased between middle‐ and late‐successional stages. The abundance of ectomycorrhizal fungi often increased significantly in the late‐successional stage of temperate and boreal forests (Clark, & St. Clair, 2011; Jiang et al., 2021; Zhang, Zhao, et al., 2022). This phenomenon is consistent with the findings of our study. The accumulation of fungal plant pathogens in the early‐successional stage may lead to increased negative plant–soil feedback, while the accumulation of ectomycorrhizal fungi in the late‐successional stage may lead to increased positive plant–soil feedback (Liang et al., 2016, 2020; Van Der Putten & Peters, 1997). Therefore, we suggest that changes in fungal functions during secondary succession in temperate forests potentially have a driving role in the succession process.

## 5 CONCLUSIONS

Our results suggest the presence of shifts in the structure and function of soil fungal communities during temperate forest secondary succession. Soil NO_3_^−^‐N and AP contents are important environmental filters in this process. Changes in fungal function in temperate forest secondary succession may potentially have a driving role in the succession process. The accumulation of fungal plant pathogens in early‐successional forests may be detrimental to seedling regeneration, thereby contributing to forest succession. At the same time, the significant accumulation of ectomycorrhizal fungi in late‐successional forests may help to maintain the dominance of established late‐succession species, suggesting that the soil microbial community shifts from promoting species turnover to promoting stability as succession progresses. These findings provide useful information to further our understanding of the response of soil fungal communities to secondary forest succession and highlight the importance of fungal community function during forest succession. It should however be taken into account that the tree species selected in this study were all symbiotic with ectomycorrhizal or arbuscular mycorrhiza and did not include species that are widely distributed in temperate zones and symbiotic with ericoid mycorrhizal; thus, our results may be limited by the mycorrhizal association of trees. Future studies should further explore the effects of differences in fungal communities on plant–soil feedback and competitive ability of different successional stage tree species through control experiments and consider including a more array of comprehensive mycorrhizal types to improve representativeness.

## AUTHOR CONTRIBUTIONS

**Xinze Geng:** Investigation (equal); methodology (equal); writing – original draft (lead). **Jincheng Zuo:** Investigation (equal); methodology (equal); visualization (lead); writing – original draft (equal). **Yunhao Meng:** Investigation (equal). **Ping Zhu:** Writing – review and editing (equal). **Yanhui Zhuge:** Methodology (equal). **Nan Wu:** Supervision (equal). **Xinfu Bai:** Conceptualization (supporting); data curation (equal); validation (supporting). **Guangyan Ni:** Investigation (supporting); methodology (supporting). **Yuping Hou:** Data curation (equal); funding acquisition (lead); writing – review and editing (equal).

## CONFLICT OF INTEREST STATEMENT

The authors declare no potential conflicts of interest.

## Supporting information


Appendix S1

## Data Availability

The original sequence data are available in the Dryad data repository: https://doi.org/10.5061/dryad.fttdz08zc.
