# Correction to “Changes in nitrogen and phosphorus availability driven by secondary succession in temperate forests shape soil fungal communities and function”

**DOI:** 10.1002/ece3.10662

**Published:** 2023-10-24

Geng, X., Zuo,J., Meng, Y., Zhuge, Y., Zhu, P., Wu, N., Bai, X., Ni, G., & Hou, Y. (2023). Changes in nitrogen and phosphorus availability driven by secondary succession in temperate forests shape soil fungal communities and function. *Ecology and Evolution*, 13, e10593. https://doi.org/10.1002/ece3.10593

The current affiliation mentioned for authors Yunhao Meng, Yanhui Zhuge, Ping Zhu, Xinfu Bai as “School of Resources and Environmental Engineering, Ludong University, Yantai, China” is wrong.

The correct affiliation should be “College of Life Sciences, Ludong University, Yantai, China”.

We apologize for this error.

