# Supplementary material for: Changes in nitrogen and phosphorus availability driven by secondary succession in temperate forests shape soil fungal communities and function
Source: Ecol Evol. 2023 Oct 9;13(10):e10593. doi: 10.1002/ece3.10593 (PMC10560873; doi:10.1002/ece3.10593)
Supplement: Supplementary file 1 — Appendix S1 [file ECE3-13-e10593-s001.docx]

Appendix

Xinze Geng, Jincheng Zuo, Yunhao Meng, Yanhui Zhuge, Ping Zhu, Nan Wu, Xinfu Bai, Guangyan Ni, and Yuping Hou

Changes in nitrogen and phosphorus availability driven by secondary succession in temperate forests shape soil fungal communities and function

Ecology and evolution

FIGURE S1


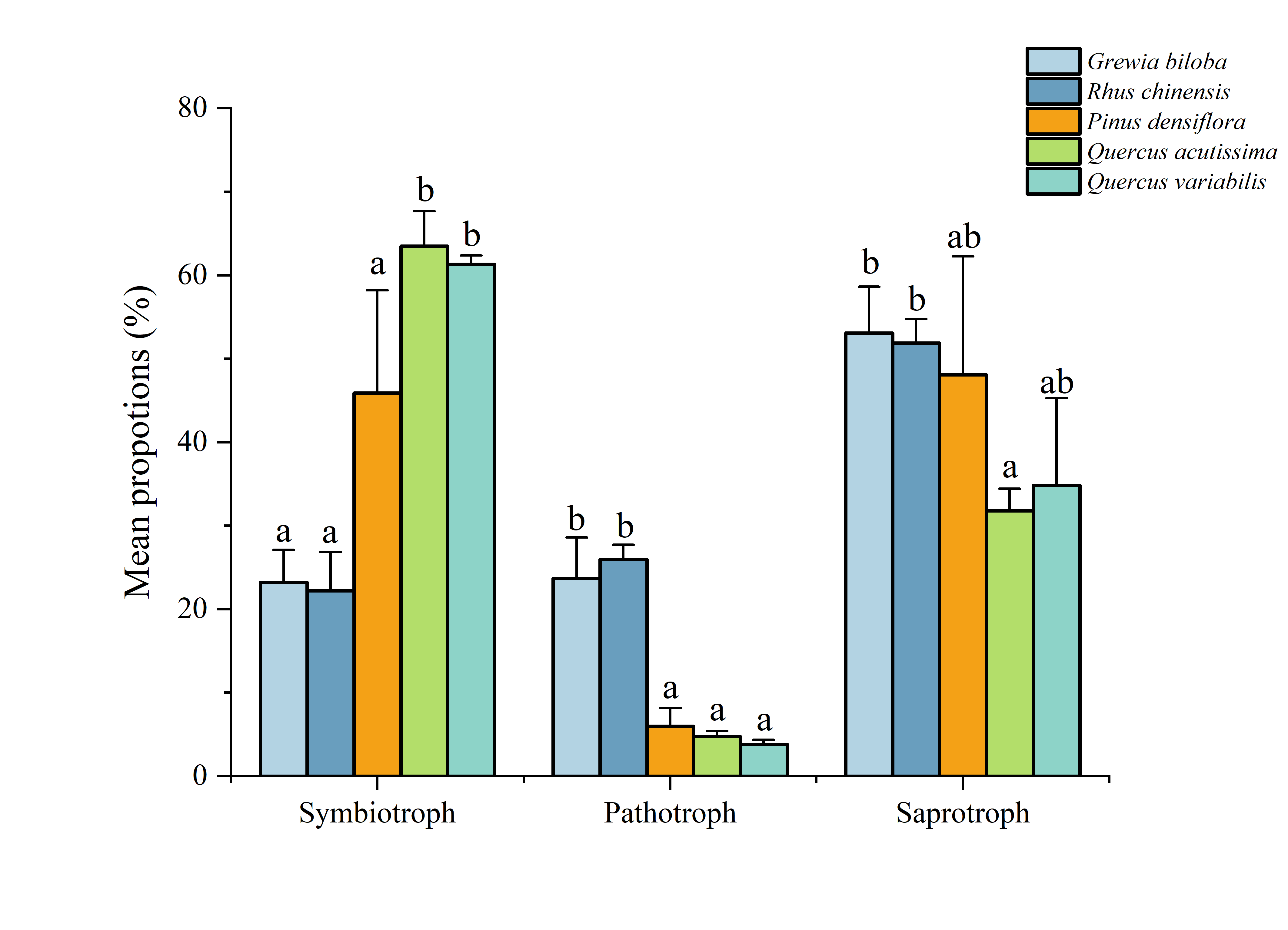


FIGURE S1 Trophic modes of fungal functional groups during the forest succession. Different letters indicate significant differences (p<0.05), no letters indicate no significant differences, and the error bars represent the standard error.

Table S1 Soil properties during the forest succession process.

| Soil properties | n | df | F-value | p-adjust | partial η² |
| --- | --- | --- | --- | --- | --- |
| pH | 15 | 4 | 6.052 | 0.01 | 0.708 |
| TC | 15 | 4 | 3.424 | 0.052 | 0.578 |
| SOC | 15 | 4 | 4.22 | 0.03 | 0.628 |
| TP | 15 | 4 | 7.565 | 0.004 | 0.752 |
| TK | 15 | 4 | 1.494 | 0.276 | 0.374 |
| TN | 15 | 4 | 3.129 | 0.065 | 0.556 |
| NH_4_^+^-N | 15 | 4 | 1.637 | 0.24 | 0.396 |
| NO_3_^-^-N | 15 | 4 | 9.772 | 0.002 | 0.796 |
| AP | 15 | 4 | 4.085 | 0.032 | 0.62 |
| AK | 15 | 4 | 8.883 | 0.003 | 0.78 |

Table S2 Fungal community richness, a-diversity, and Shannon evenness during the forest succession process.

| Index | n | df | F-value | p-adjust | partial η² |
| --- | --- | --- | --- | --- | --- |
| Chao1 | 15 | 4 | 20.195 | ＜0.001 | 0.891 |
| Shannon | 15 | 4 | 20.467 | ＜0.001 | 0.891 |
| Shannon evenness | 15 | 4 | 18.183 | ＜0.001 | 0.879 |

Table S3 Differences between fungal communities at the phylum and genus levels during forest succession.

| Phylum and genus | n | df | F-value | p-adjust | partial η² |
| --- | --- | --- | --- | --- | --- |
| Basidiomycota | 15 | 4 | 59.987 | ＜0.001 | 0.96 |
| Ascomycota | 15 | 4 | 14.794 | 0.001 | 0.855 |
| Mortierellomycota | 15 | 4 | 8.993 | 0.02 | 0.782 |
| *Russula* | 15 | 4 | 9.932 | 0.002 | 0.799 |
| *Mortierella* | 15 | 4 | 9.013 | 0.02 | 0.783 |
| *Penicillium* | 15 | 4 | 3.539 | 0.004 | 0.586 |

Table S4 Trophic modes of fungal functional groups during forest succession.

| Functional groups | n | df | F-value | p-adjust | partial η² |
| --- | --- | --- | --- | --- | --- |
| Symbiotroph | 15 | 4 | 10.013 | 0.002 | 0.8 |
| Ectomycorrhiza | 15 | 4 | 21.962 | ＜0.001 | 0.898 |
| Arbuscular mycorrhiza | 15 | 4 | 5.616 | 0.012 | 0.692 |
| Ericoid mycorrhiza | 15 | 4 | 13.617 | ＜0.001 | 0.845 |
| Pathotroph | 15 | 4 | 22.948 | ＜0.001 | 0.902 |
| Plant pathogen | 15 | 4 | 19.089 | ＜0.001 | 0.884 |
| Animal pathogen | 15 | 4 | 16.74 | ＜0.001 | 0.87 |
| Saprotroph | 15 | 4 | 3.529 | 0.048 | 0.585 |
| Dung saprotroph | 15 | 4 | 8.717 | 0.003 | 0.777 |
| Leaf saprotroph | 15 | 4 | 2.982 | 0.073 | 0.544 |
| Plant saprotroph | 15 | 4 | 1.923 | 0.183 | 0.435 |
| Soil saprotroph | 15 | 4 | 6.211 | 0.009 | 0.713 |
| Wood saprotroph | 15 | 4 | 10.906 | 0.001 | 0.814 |
